# Efficacy and safety of sintilimab plus bevacizumab with or without transarterial chemoembolization for unresectable hepatocellular carcinoma: a propensity score-matched retrospective cohort study

**DOI:** 10.3389/fimmu.2026.1928849

**Published:** 2026-10-26

**Authors:** Zizhuo Wang, Bailu Wu, Chunshu Wu, Hongquan Wu, Shihao Zhao, Shuwen Ye, Xiaodong Li, Pengchao Zhan, Xin Li, Zhen Li

**Affiliations:** 1 Department of Interventional Radiology, The First Affiliated Hospital of Zhengzhou University, Zhengzhou, Henan, China; 2 Henan Provincial Minimally Invasive Interventional Tumor Engineering and Technology Research Center, Zhengzhou, Henan, China; 3 Key Laboratory of Innovative and Translational Interventional Oncology Technology in Zhengzhou, Zhengzhou, Henan, China; 4 Department of Radiology, Third People’s Hospital of Henan Province, Zhengzhou, Henan, China

**Keywords:** bevacizumab, hepatocellular carcinoma, propensity score matching, sintilimab, transarterial chemoembolization

## Abstract

**Background:**

To compare the efficacy and safety of transarterial chemoembolization (TACE) plus sintilimab and bevacizumab (triple therapy) versus sintilimab and bevacizumab alone (dual therapy) in unresectable hepatocellular carcinoma (uHCC).

**Methods:**

This single-center retrospective cohort study enrolled consecutive patients with uHCC who received triple or dual therapy between October 2021 and May 2025. Propensity score matching (PSM) was performed using logistic regression (1:1 nearest-neighbor, caliper: 0.02 SD). The primary endpoint was progression-free survival (PFS). The secondary endpoints included objective response rate (ORR), disease control rate (DCR), overall survival (OS), and safety. An exploratory prognostic score for PFS was developed in the matched cohort and used to define risk strata for hypothesis-generating analyses.

**Results:**

After PSM, 40 patients were included in each group. The triple therapy group achieved a longer median PFS than the dual-therapy group (13.11 vs. 8.57 months; log-rank *P* = 0.026). The median OS was not reached (log-rank *P* = 0.20). The ORR (75.0% vs. 60.0%; *P* = 0.233), DCR (95.0% vs. 82.5%; *P* = 0.154), and radiographic downstaging rate (17.5% vs. 5.0%; *P* = 0.154) were numerically higher with triple therapy. The prognostic model demonstrated that, median PFS was 14.46 months, 11.50 months, and 5.62 months in the low-, intermediate-, and high-risk groups, respectively (log-rank *P* < 0.001). Risk-stratified HR estimates were numerically lowest in the intermediate-risk group; however, the formal treatment-by-risk-group interaction was not statistically significant (*P* for interaction = 0.879). Grade 3–4 adverse events were numerically more frequent with triple therapy (37.5% vs. 22.5%; *P* = 0.222), with no treatment-related deaths.

**Conclusions:**

In PSM-matched uHCC patients, TACE plus sintilimab-bevacizumab was associated with longer PFS, with generally manageable toxicity but a numerically higher incidence of grade 3–4 adverse events. Exploratory risk-stratified analyses generated hypotheses regarding potential differences in treatment benefit across prognostic strata.

## Introduction

Primary liver cancer ranks sixth in global incidence and third in cancer-related mortality worldwide (1). Hepatocellular carcinoma (HCC) accounts for 75–85% of primary liver cancer cases and represents the predominant histological subtype. The prognosis of HCC remains dismal, with more than half of the patients presenting at an intermediate or advanced stage at diagnosis (2), precluding curative treatments such as surgical resection, liver transplantation, or ablation (3). The five-year survival rate was approximately 12.1% (4). For advanced-stage disease, international guidelines recommend systemic therapy as the first-line treatment (3, 5); however, objective response rates remain suboptimal, underscoring the urgent need for novel combination strategies. Transarterial chemoembolization (TACE) has historically been the standard locoregional treatment for intermediate-stage unresectable HCC (uHCC), but recent Asian guidelines have expanded its indications to encompass advanced-stage disease as a recognized standard-of-care option (6, 7). This expansion creates a compelling rationale for combining TACE with systemic agents and broadens the therapeutic landscape. The integration of TACE with targeted therapies and immune checkpoint inhibitors has achieved substantial progress in the management of uHCC, significantly improving patient outcomes (8, 9).

Surgical resection remains a potentially curative modality for achieving long-term survival or cure of HCC (5, 10). The application of combination regimens as conversion therapy in uHCC has attracted increasing interest, with the aim of achieving tumor downstaging and providing patients with initially unresectable or borderline-resectable tumors the opportunity to undergo curative-intent resection (11). Nevertheless, conversion therapy for uHCC remains controversial, and has not been widely implemented in routine clinical practice (12). Prior investigations have demonstrated the feasibility of conversion therapy combining tyrosine kinase inhibitors (TKIs) and anti-PD-1 antibodies, achieving an R0 resection rate of 15.9% and a pathological response rate of 60% (13). The addition of TACE may further augment the synergistic effect of conversion (10, 14, 15). Notably, a prior study reported that locoregional intervention combined with bevacizumab and sintilimab yielded a higher conversion rate than locoregional intervention combined with lenvatinib and sintilimab (57.1% vs. 26.0%; *P* = 0.043), although no significant differences in PFS or OS were observed between the two regimens (10). However, direct comparative evidence on tumor response and radiographic downstaging with TACE-based combination therapy versus systemic immunotherapy–targeted therapy doublets remains limited.

Following the regulatory approval of sintilimab plus bevacizumab as a first-line regimen in China, this combination has been widely adopted in clinical practice. However, treatment outcomes remain suboptimal in a subset of patients, and the additional integration of locoregional therapy holds promise for further improving patient benefit. The antitumor efficacy, radiographic downstaging rate, and long-term outcomes of sintilimab plus bevacizumab with or without TACE in uHCC have not been adequately characterized. Therefore, this retrospective study assessed the treatment response, survival, and safety in patients with uHCC who received either TACE combined with sintilimab and bevacizumab or sintilimab and bevacizumab alone.

## Methods

### Patient selection

This retrospective cohort study enrolled consecutive patients with uHCC who received either TACE combined with sintilimab and bevacizumab (triple therapy group) or sintilimab and bevacizumab alone (dual therapy group) at the First Affiliated Hospital of Zhengzhou University, between October 2021 and May 2025. The study was approved by the Institutional Review Board of the First Affiliated Hospital of Zhengzhou University (Approval No. 2024-KY-1991-003). Given the retrospective nature of this study, the requirement for informed consent was waived. Because this was a retrospective real-world study, treatment allocation was not randomized or governed by a prespecified study protocol. BCLC stage was a major clinical consideration in determining whether TACE was incorporated into the treatment strategy. The final treatment decision was individualized by the treating team according to the distribution and intrahepatic burden of the tumors, hepatic functional reserve, technical feasibility and anticipated benefit of TACE, contraindications to locoregional treatment, and patient preference. The reasons underlying the decision to add or not add TACE were not prospectively standardized and could not be fully reconstructed from the retrospective medical records.

The inclusion criteria were as follows (1): age ≥18 years (2); Eastern Cooperative Oncology Group Performance Status (ECOG PS) score of 0–1 (3); histologically or clinically confirmed HCC diagnosis in accordance with the American Association for the Study of Liver Diseases (AASLD) criteria (4); deemed unresectable by the treating clinician following multidisciplinary team (MDT) evaluation (The MDT included specialists in hepatobiliary surgery, interventional radiology, radiology, and hepatology. Unresectability was determined on the basis of tumor extent and distribution, vascular invasion or extrahepatic spread, anticipated future liver remnant, hepatic functional reserve, and the feasibility of achieving an R0 resection.) (5); Barcelona Clinic Liver Cancer (BCLC) stage A–C (6); no prior local or systemic anticancer therapy for HCC (7); Child-Pugh class A or B; and (8) at least one measurable target lesion per the modified Response Evaluation Criteria in Solid Tumors (mRECIST). Patients were excluded if they received other tumor-related treatments, such as hepatic arterial infusion chemotherapy (HAIC) or radiation therapy, during the combination therapy period, had concurrent malignancies, had incomplete or missing clinical or imaging data, or were lost to follow-up for more than three months.

### TACE procedure

All the patients in the triple therapy group underwent drug-eluting bead TACE (DEB-TACE). Drug-eluting beads with diameters of 100–300 µm or 300–500 µm (2–4 mL per session; DC Beads, Biocompatibles, Farnham, UK; Callispheres beads, Jiangsu Hengrui Medicine Co., Ltd., China; or HepaSpheres, Biosphere Medical, Inc., South Jordan, UT, USA) were used. TACE with drug-eluting beads was performed with epirubicin or doxorubicin (maximum dose, 100 mg), without a pre-specified bead size or chemotherapy dose protocol. Following successful common femoral artery access via the Seldinger technique and placement of an arterial introducer sheath, a 5F visceral catheter was advanced into the proper hepatic artery. Selective digital subtraction arteriography was performed to identify the hypervascular tumors and their feeding arteries. A 2.7F coaxial microcatheter (Progreat, Terumo, Tokyo, Japan) was then advanced into the tumor-feeding artery under fluoroscopic guidance and the bead-drug emulsion was manually infused. The need for additional embolization was determined at the operator’s discretion according to tumor burden, vascularity, arterial anatomy, angiographic findings, and prior treatment response. Additional embolization was performed if the desired angiographic endpoint was not achieved, which was defined as complete stasis of antegrade flow in the tumor-feeding artery or complete disappearance of tumor staining on arteriography.

Subsequent on-demand TACE was performed in cases of residual viable tumors or intrahepatic recurrence on contrast-enhanced CT or MRI. The decision to repeat TACE was made by the MDT based on imaging findings, with a minimum interprocedural interval of 4–6 weeks.

### Sintilimab and bevacizumab

Patients in both groups received sintilimab (Daboshu^®^, Innovent Biologics, Suzhou, China; 200 mg intravenously over 60 min, every 3 weeks [q3w]) followed by a bevacizumab biosimilar (BYVASDA^®^, Innovent Biologics, China; 7.5 mg/kg intravenously over 90 min for the first infusion, 60 min for the second infusion, and 30 min thereafter if well tolerated, q3w). In the triple therapy group, systemic therapy was initiated within three days of the first TACE session. The maximum duration of systemic treatment was 24 months, or until disease progression, death, intolerable toxicity, initiation of a new antineoplastic regimen, or any other protocol-specific reason for discontinuation.

### Study endpoints

Tumor response was evaluated according to mRECIST. The primary endpoint was progression-free survival (PFS), defined as the time from treatment initiation to the first documented disease progression or death from any cause, whichever occurred first. Treatment initiation was defined as the date of the first TACE procedure in the triple-therapy group and the first administration of sintilimab plus bevacizumab in the dual-therapy group. Secondary endpoints included (1) objective response rate (ORR), defined as the proportion of patients achieving a best overall response of complete response (CR) or partial response (PR) according to mRECIST, without requiring confirmation on subsequent imaging (2); disease control rate (DCR), defined as the proportion of patients achieving CR, PR, or stable disease (SD) (3); time to response (TTR), defined as the interval from treatment initiation to the first documented objective response according to mRECIST among responding patients, without requiring confirmation on subsequent imaging (4); duration of response (DOR), defined as the interval from the first documented response to first recorded disease progression or death from any cause in responding patients (5); overall survival (OS), defined as the time from treatment initiation to death from any cause or last follow-up (6); radiographic downstaging rate, defined as the proportion of patients whose CNLC stage at the assessment corresponding to the best overall radiological response was lower than their baseline CNLC stage. A decrease of at least one CNLC stage category, including a change between substages, was considered downstaging. Surgical resectability and subsequent surgical resection were not required to meet this endpoint; and (7) safety, graded according to the Common Terminology Criteria for Adverse Events (CTCAE) version 5.0.

### Data collection and follow-up

The baseline characteristics and clinical parameters were retrospectively extracted from the electronic medical records of all eligible patients. Systematic radiological surveillance incorporating contrast-enhanced abdominal CT or MRI and chest CT was conducted at baseline, every 6 weeks during the initial 12-month therapeutic phase, and every 12 weeks thereafter, until disease progression or death. Independent radiographic evaluations were performed in a dual-blinded fashion by two board-certified radiologists (Z. Li and B. Wu) with more than 10 years of experience in hepatobiliary imaging. Two radiologists independently reviewed the imaging data, with each reader blinded to treatment allocation, the assessments of the other radiologist, and the clinical outcome data. In cases of discordant assessments between the two readers, a third senior radiologist adjudicated to achieve a consensus. Radiographic downstaging rate reflected radiographic stage reduction and should not be interpreted as successful conversion to surgical resectability. Data on subsequent surgical resection were not collected in the study dataset. Therefore, the number and proportion of patients who ultimately underwent resection could not be reported. The data cut-off date for this analysis was April 2026.

### Statistical analysis

Categorical and continuous variables were summarized as counts (%) and medians (ranges), respectively, and compared using appropriate tests detailed in the Supplementary Method 1. Propensity scores were estimated using baseline BCLC stage alone, reflecting a parsimonious strategy based on its prognostic and treatment-selection relevance in this small cohort. Patients were matched 1:1 using nearest-neighbor matching without replacement and a caliper of 0.02 standard deviations of the propensity score. Balance was assessed using absolute standardized mean differences, with 0.10 as a descriptive reference.

Time-to-event outcomes were estimated using the Kaplan–Meier method with 95% confidence intervals (CIs) and compared using log-rank tests. Fixed-time PFS and DOR probabilities were compared using two-sided Wald tests on the log[−log(S(t))] scale with Greenwood variances, without matched-pair covariance or multiplicity adjustment. Cox regression was used for PFS prognostic and subgroup analyses, and logistic regression for ORR. Variables with univariable P ≤ 0.10 entered the multivariable PFS model. An exploratory prognostic score was developed. Risk strata were defined using sample-specific tertiles of the linear predictor. Discrimination was assessed using Harrell’s C-index, with 1,000 bootstrap resamples for internal validation of the fixed three-variable model. Treatment-by-risk-group interaction was evaluated using a likelihood-ratio test. Detailed procedures and censoring rules are provided in the **Supplementary Method 1**.

Analyses used R 4.5.2 and GraphPad Prism 10.0. All tests were two-sided, with P < 0.05 considered statistically significant.

## Results

### Patient characteristics

Prior to PSM, 84 and 40 patients were enrolled in the dual and triple therapy groups, respectively (**Supplementary Table 1**). Following 1:1 nearest-neighbor PSM, 40 patients were retained in each treatment group (**Figure 1**). After matching, no baseline characteristic showed a statistically significant between-group difference (**Table 1**). Absolute standardized mean differences indicated residual imbalance in several other measured covariates, as detailed in **Supplementary Table 2** and **Supplementary Figure 1**. The treatment cycle data after PSM are shown in **Supplementary Table 3**. In the triple therapy group, the median number of TACE sessions was 2.0 (range, 1.0–3.0). Regarding systemic treatment cycles, the triple therapy group received significantly more sintilimab cycles (median, 13.0 vs. 8.0; *P* = 0.011) and bevacizumab cycles (median, 13.0 vs. 6.5; *P* < 0.001) than the dual therapy group.

**Figure 1:**
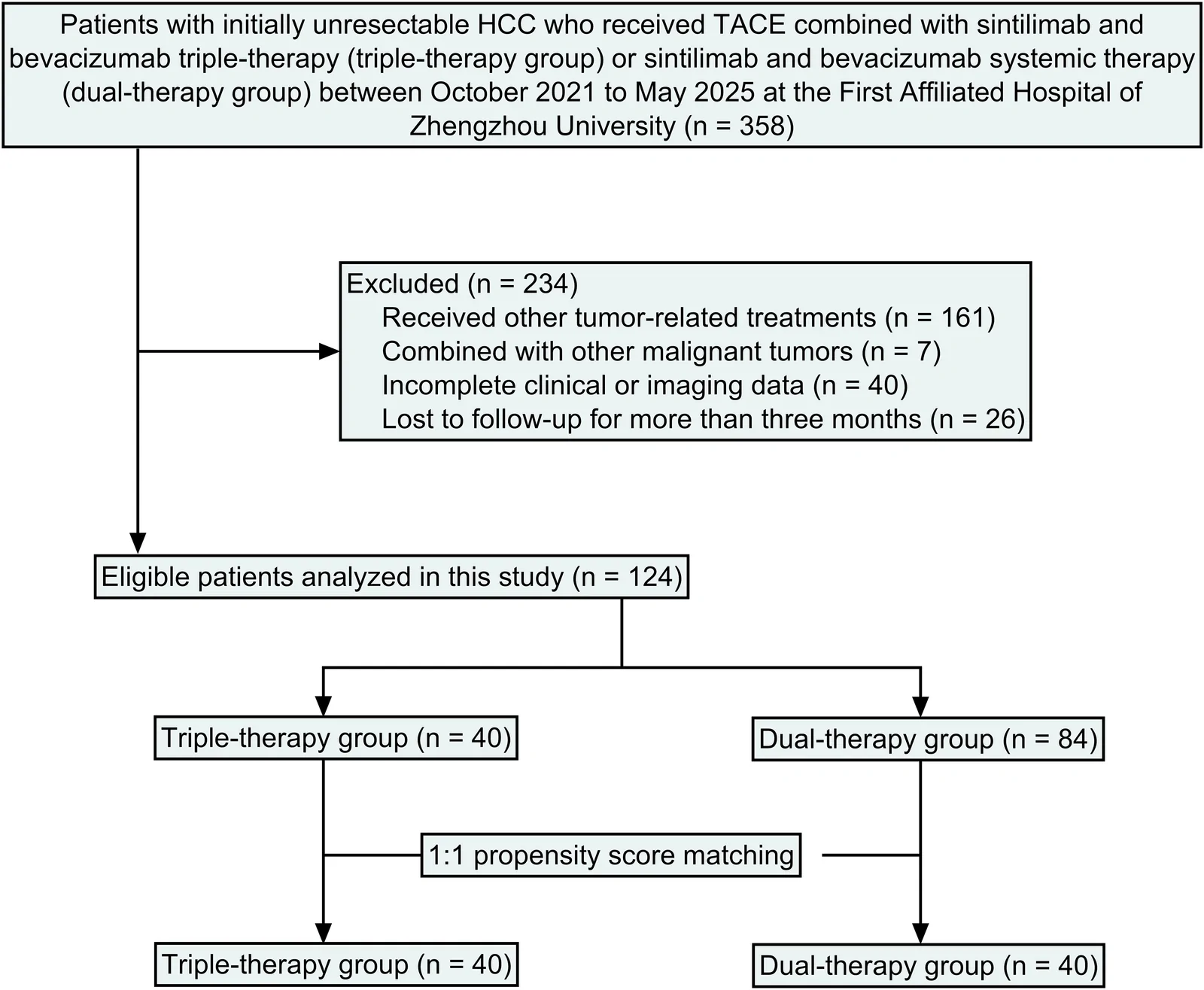
Flowchart for patient recruitment in two cohort groups. The diagram illustrates the selection process of patients with unresectable hepatocellular carcinoma who received either triple therapy (TACE + sintilimab + bevacizumab) or dual therapy (sintilimab + bevacizumab alone), including inclusion and exclusion criteria, and the final matched cohort after 1:1 propensity score matching.

**Table 1:** Patient baseline characteristics after matching.

Characteristics, n (%)	Dual-therapy group (n = 40)	Triple-therapy group (n = 40)	*P*-value
Gender			0.056
Female	5 (12.5%)	12 (30.0%)	
Male	35 (87.5%)	28 (70.0%)	
Age, years			0.172
<50	11 (27.5%)	6 (15.0%)	
≥50	29 (72.5%)	34 (85.0%)	
PVTT			0.271
Vp 0	16 (40.0%)	9 (22.5%)	
Vp 1	0 (0.0%)	3 (7.5%)	
Vp 2	9 (22.5%)	10 (25.0%)	
Vp 3	8 (20.0%)	8 (20.0%)	
Vp 4	7 (17.5%)	10 (25.0%)	
HVTT			0.284
absent	33 (82.5%)	29 (72.5%)	
present	7 (17.5%)	11 (27.5%)	
Extrahepatic spread			0.626
absent	29 (72.5%)	27 (67.5%)	
present	11 (27.5%)	13 (32.5%)	
CNLC stage			0.972
2a	1 (2.5%)	1 (2.5%)	
2b	4 (10.0%)	4 (10.0%)	
3a	24 (60.0%)	22 (55.0%)	
3b	11 (27.5%)	13 (32.5%)	
BCLC			1.000
B	5 (12.5%)	5 (12.5%)	
C	35 (87.5%)	35 (87.5%)	
Tumor size^*^, cm	7.80 (3.70–18.00)	7.55 (2.40–14.70)	0.213
Tumor number			0.371
≤3	18 (45.0%)	22 (55.0%)	
>3	22 (55.0%)	18 (45.0%)	
Up-to-7			0.675
≤7	2 (5.0%)	4 (10.0%)	
>7	38 (95.0%)	36 (90.0%)	
AFP, ng/mL			0.653
<400	21 (52.5%)	23 (57.5%)	
≥400	19 (47.5%)	17 (42.5%)	
Etiology			1.000
HCV	4 (10.0%)	3 (7.5%)	
HBV	35 (87.5%)	35 (87.5%)	
None	1 (2.5%)	2 (5.0%)	
ALBI grade			0.369
1	16 (40.0%)	10 (25.0%)	
2	23 (57.5%)	29 (72.5%)	
3	1 (2.5%)	1 (2.5%)	
Child-Pugh			0.133
A	26 (65.0%)	32 (80.0%)	
B	14 (35.0%)	8 (20.0%)	
ECOG PS			0.491
0	14 (35.0%)	17 (42.5%)	
1	26 (65.0%)	23 (57.5%)	
NLR^*^	2.37 (0.66–12.48)	2.70 (0.76–9.06)	0.570

* Median (range). PVTT, Portal vein tumor thrombus; HVTT, Hepatic vein tumor thrombus; CNLC, China Liver Cancer Staging; BCLC, Barcelona Clinic Liver Cancer; AFP, Alpha-fetoprotein; ALBI, Albumin-Bilirubin grade; ECOG PS, Eastern Cooperative Oncology Group Performance Status; NLR, Neutrophil-to-Lymphocyte Ratio.

### Tumor response and downstaging

The post-PSM tumor response data are presented in **Table 2**. The ORR was numerically higher in the triple therapy group than in the dual therapy group (75.0% vs. 60.0%), although the difference was not statistically significant (*P* = 0.233). DCR was also numerically higher in the triple therapy group (95.0% vs. 82.5%), without a statistically significant between-group difference (*P* = 0.154). Multivariable logistic regression identified tumor number >3 (odds ratio [OR] = 0.25; 95% CI: 0.08–0.71; *P* = 0.011) and AFP ≥400 ng/mL (OR = 0.32; 95% CI: 0.11–0.87; *P* = 0.028) as independent negative predictors of ORR (**Supplementary Table 4**).

**Table 2:** Tumor response and radiographic downstaging during treatment after matching.

Variables, n (%)	Dual-therapy group (n = 40)	Triple-therapy group (n = 40)	*P*-value
ORR	24 (60.0%)	30 (75.0%)	0.233
DCR	33 (82.5%)	38 (95.0%)	0.154
Best overall response			0.086
CR	2 (5.0%)	4 (10.0%)	
PR	22 (55.0%)	26 (65.0%)	
SD	9 (22.5%)	8 (20.0%)	
PD	7 (17.5%)	2 (5.0%)	
Radiographic downstaging	2 (5.0%)	7 (17.5%)	0.154
CNLC stage at the best radiological response assessment			0.647
Ia	0 (0.0%)	4 (10.0%)	
IIa	3 (7.5%)	4 (10.0%)	
IIb	4 (10.0%)	3 (7.5%)	
IIIa	23 (57.5%)	17 (42.5%)	
IIIb	10 (25.0%)	12 (30.0%)	

Radiographic downstaging was defined as a decrease of at least one CNLC stage category from baseline to the assessment corresponding to the best overall radiological response, irrespective of surgical resectability or subsequent resection. ORR, Objective response rate; DCR, Disease control rate; CR, Complete response; PR, Partial response; SD, Stable disease; PD, Progressive disease; CNLC, China Liver Cancer Staging.

No statistically significant between-group differences were observed with respect to the best overall response (*P* = 0.086). In the triple therapy group, the CR, PR, SD, and PD rates were 10.0%, 65.0%, 20.0%, and 5.0%, respectively; the corresponding rates in the dual therapy group were 5.0%, 55.0%, 22.5%, and 17.5%, respectively. Waterfall plots depicting the best percentage change from baseline in target lesion size and swimmer plots illustrating the longitudinal trajectory of patient events in both matched groups are presented in **Figures 2A–D**.

**Figure 2:**
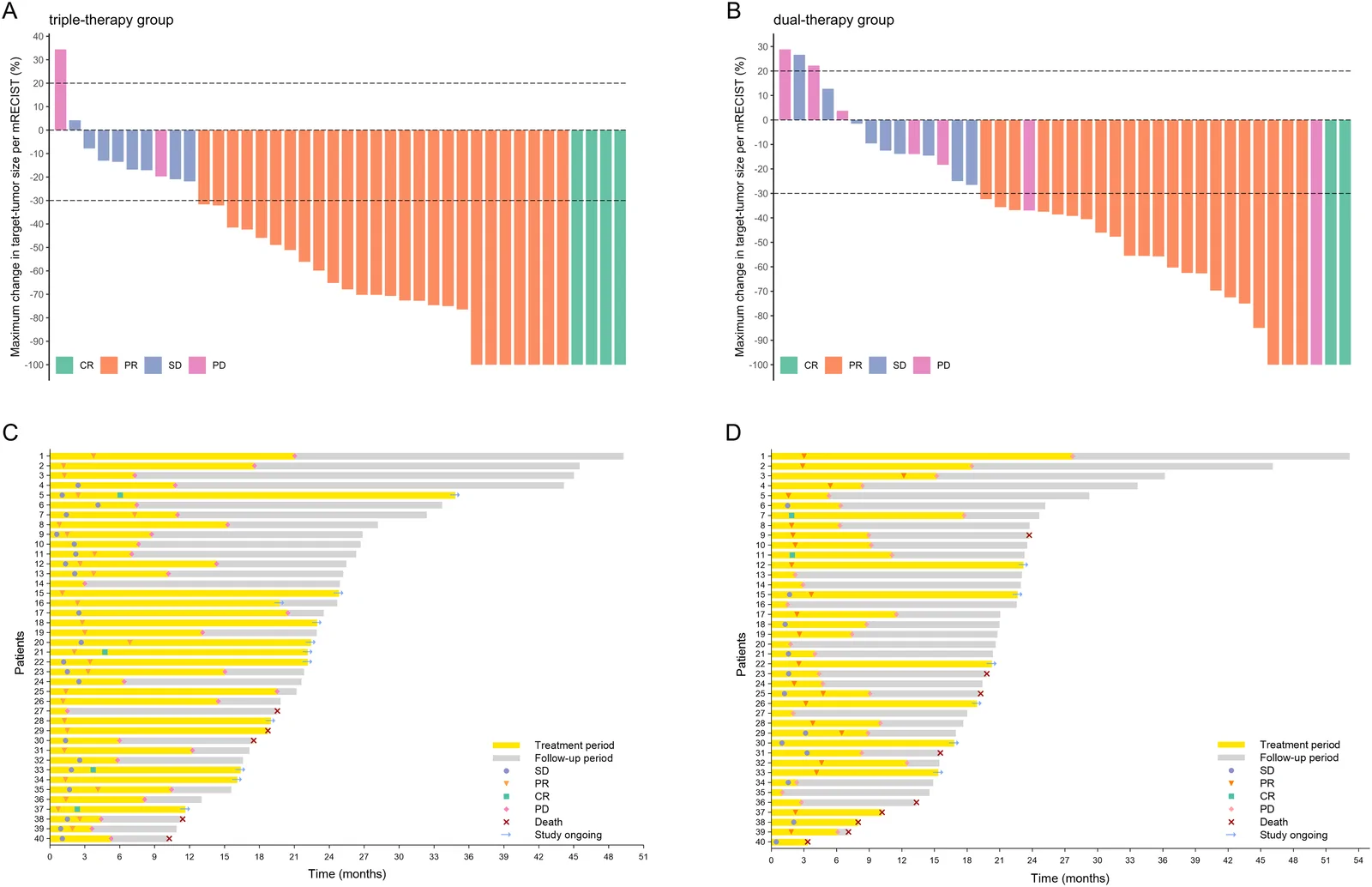
Waterfall plots showing best percentage change from baseline in target lesion size assessed by mRECIST criteria for the triple therapy group **(A)** and dual therapy group **(B)**. Swimmer plots illustrating tumor response and event occurrence for each patient during treatment and follow-up in the triple therapy group **(C)** and dual therapy group **(D)**.

Radiographic downstaging occurred in 7 of 40 patients (17.5%) in the triple-therapy group and 2 of 40 patients (5.0%) in the dual-therapy group (*P* = 0.154; **Table 2**). The distribution of CNLC stages at the best radiological response assessment did not differ significantly between groups (*P* = 0.647). These results describe radiographic stage reduction and do not establish conversion to surgical resectability. Subsequent surgical resection data were not collected.

### Survival outcomes and efficacy

After matching, the median follow-up duration was 22.97 months (range, 10.25–49.28 months) in the triple therapy group and 22.57 months (range, 3.35–53.16 months) in the dual therapy group (*P =* 0.510). The triple therapy group achieved a significantly longer median PFS (**Figure 3A**) of 13.11 months (95% CI: 10.18–19.50) compared with 8.57 months (95% CI: 6.31–11.10) in the dual therapy group (log-rank *P =* 0.026). The 6-, 9-, and 12-month PFS rates were 82.5% vs. 67.5% (*P* = 0.129), 65.0% vs. 42.1% (*P* = 0.043), and 55.0% vs. 26.3% (*P* = 0.010), respectively. The median OS (**Figure 3B**) was not reached in either group (log-rank *P =* 0.20).

**Figure 3:**
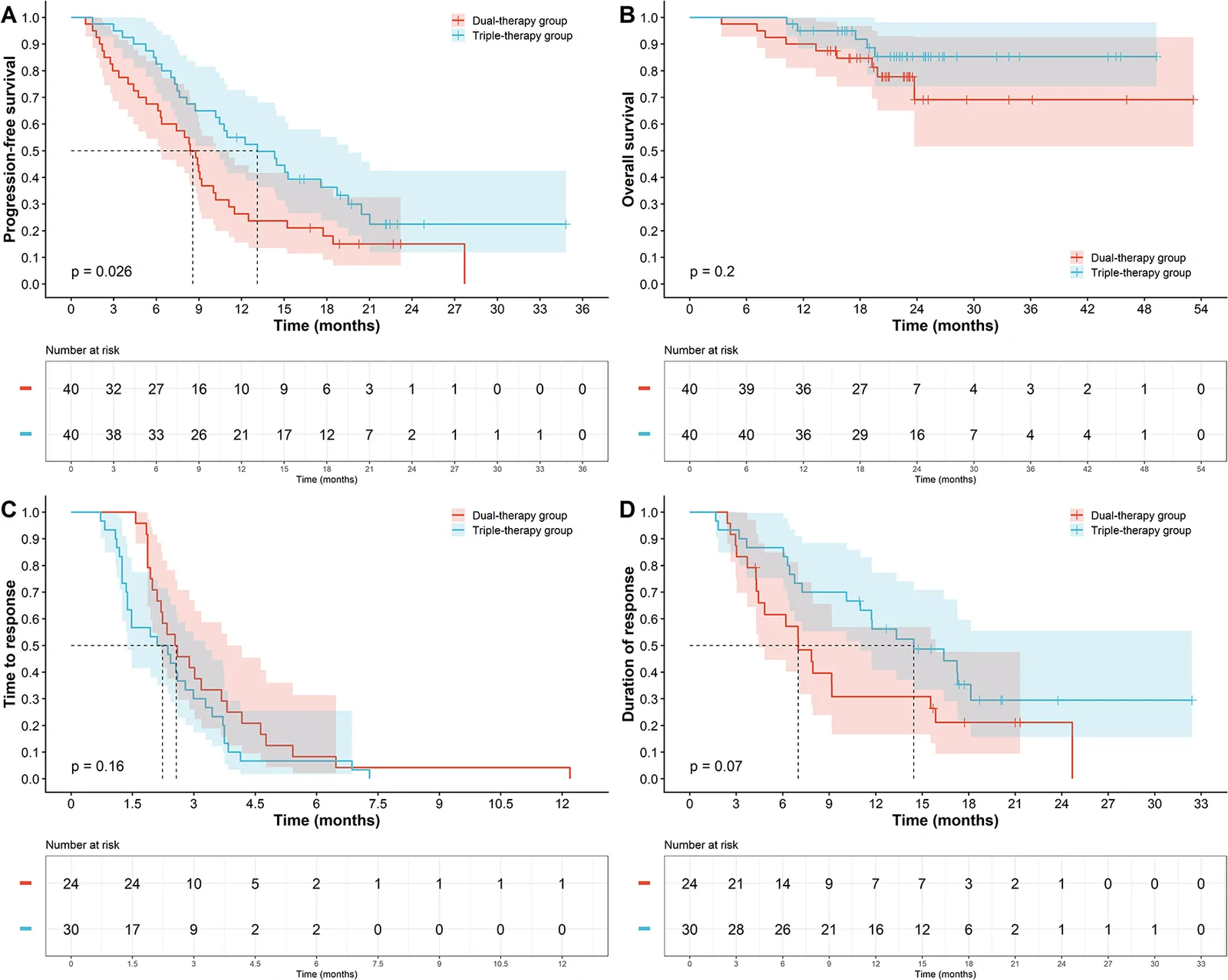
Kaplan-Meier analysis of progression-free survival **(A)**, overall survival **(B)**, time to response **(C)**, and duration of response **(D)** in the two treatment groups.

Among patients who achieved an objective response (**Figure 3C, D**), the median TTR was 2.23 months (95% CI: 1.38–3.29) in the triple therapy group and 2.57 months (95% CI: 2.20–3.81) in the dual therapy group (log-rank *P =* 0.16). The median DOR was 14.5 months (95% CI: 11.01–not reached) in the triple therapy group versus 7.0 months (95% CI: 4.44–15.9) in the dual therapy group (log-rank *P =* 0.07). Among responders, the 6- and 12-month DOR rates were 86.7% vs. 61.6% (*P* = 0.043) and 56.1% vs. 30.8% (*P* = 0.066), respectively.

In the multivariable Cox proportional hazards analysis for PFS (**Supplementary Table 5**), sex violated the proportional hazards assumption in Schoenfeld residual testing and was therefore modeled as a time-varying covariate in a revised analysis. Extrahepatic metastasis (HR = 1.85; 95% CI: 1.04–3.28; Cox model Wald *P =* 0.035) and tumor number >3 (HR = 1.95; 95% CI: 1.12–3.40; Cox model Wald *P =* 0.019) were identified as independent adverse prognostic factors for PFS. PFS subgroup analyses (**Figure 4**) showed that triple therapy was associated with a lower risk of progression in the overall matched cohort (HR, 0.57; 95% CI, 0.35–0.94; Cox model Wald *P* = 0.028). Point estimates also generally favored triple therapy in several subgroups, although most subgroup findings were imprecise and exploratory.

**Figure 4:**
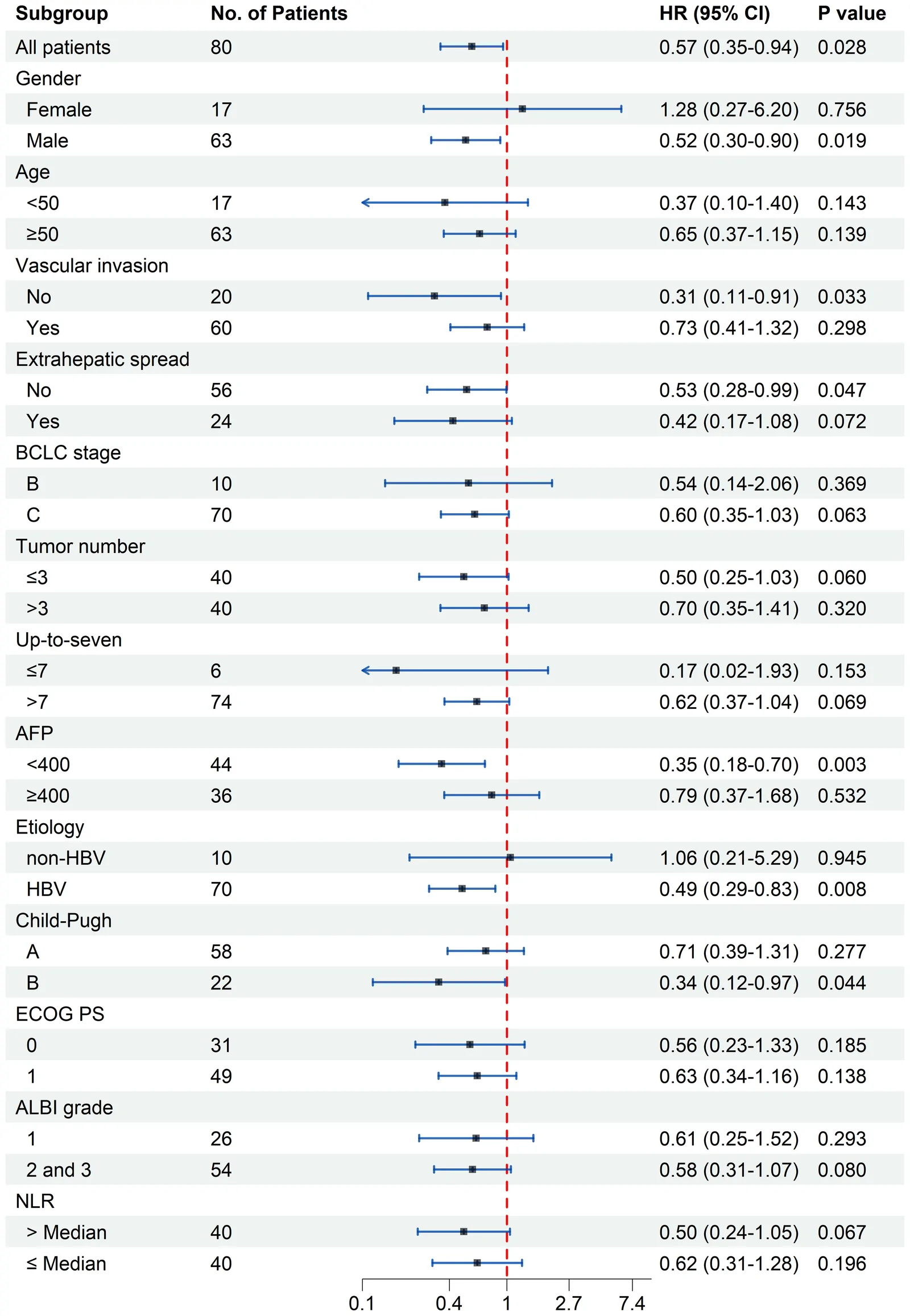
Subgroup Analysis of Progression-Free Survival. Forest plot showing the treatment effect (triple therapy vs. dual therapy) on progression-free survival across prespecified subgroups, including age, sex, AFP level, tumor number, vascular invasion, extrahepatic spread, Child-Pugh class, and ALBI grade. Hazard ratios, 95% confidence intervals, and corresponding P values were derived from univariable Cox proportional hazards models using Wald tests.

### Exploratory development of the PFS prognostic model

Two independent prognostic factors identified by multivariable Cox regression (extrahepatic metastasis and tumor number >3) were incorporated into the prognostic model. Child-Pugh class B demonstrated a marginal association with PFS in multivariable analysis (*P =* 0.060); given its established clinical relevance and contribution to overall model performance, it was included in the final nomogram (**Figure 5A**). The exploratory model showed modest discrimination, with an apparent C-index of 0.639 and an optimism-corrected C-index of 0.622.

**Figure 5:**
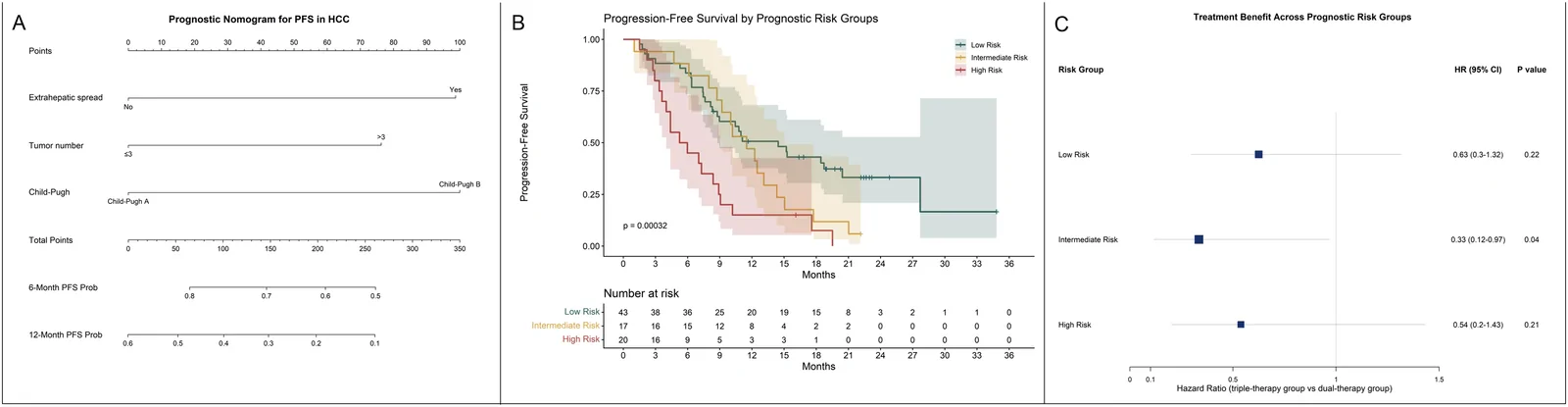
Exploratory prognostic nomogram and risk-stratified PFS analyses. **(A)** Prognostic nomogram for predicting 6-month and 12-month progression-free survival based on extrahepatic spread, tumor number >3, and Child-Pugh class B. **(B)** Kaplan-Meier curves of PFS according to low- (n = 43), intermediate- (n =17), and high-risk (n =20) groups derived from the nomogram. **(C)** Forest plot showing the treatment effect (TACE plus sintilimab and bevacizumab vs. sintilimab and bevacizumab alone) on progression-free survival within each risk stratum. Within each prognostic risk stratum, the treatment effect of triple therapy versus dual therapy on PFS was estimated using a univariable Cox proportional hazards model. The overall treatment-by-risk-group interaction was evaluated using a two-degree-of-freedom likelihood-ratio test (*P* for interaction = 0.879). Hazard ratios, 95% confidence intervals, and corresponding P values were derived from the Cox model, with P values calculated using Wald tests. The prognostic model was developed in the 80-patient matched cohort and has not been externally validated; all risk-stratified analyses should therefore be considered exploratory.

Risk stratification was performed by computing the linear predictor (LP) score for each patient using the regression coefficients of the prognostic factors: LP *=* (0.5699 × extrahepatic metastasis) + (0.4403 × tumor number >3) + (0.5773 × Child-Pugh class B), where each variable was scored as 1 if present and 0 otherwise. The 80 PSM-matched patients were subsequently stratified into low-, intermediate-, and high-risk groups based on the LP tertile cutoffs.

Median PFS was 14.46 months (95% CI: 8.77–not reached), 11.50 months (95% CI: 9.20–15.05), and 5.62 months (95% CI: 4.01–9.07) in the low-, intermediate-, and high-risk groups, respectively. Log-rank test revealed a statistically significant difference in survival across the three risk strata (*P* < 0.001; **Figure 5B**).

In exploratory risk-stratified analyses, the estimated HRs for triple therapy versus dual therapy were 0.63 (95% CI: 0.30–1.32; nominal *P* = 0.216), 0.33 (95% CI: 0.12–0.97; nominal *P* = 0.043), and 0.54 (95% CI: 0.20–1.43; nominal *P* = 0.215) in the low-, intermediate-, and high-risk groups, respectively (**Figure 5C**). Although the point estimates were below 1.0 in all three strata and were numerically lowest in the intermediate-risk group, the overall treatment-by-risk-group interaction was not statistically significant (likelihood-ratio χ² = 0.258, df = 2; *P* for interaction = 0.879). Therefore, there was no statistical evidence that the association between treatment and PFS differed across the three risk strata. The finding in the intermediate-risk group should be considered hypothesis-generating rather than evidence of a subgroup-specific treatment benefit.

### Safety

Treatment-emergent adverse events (TEAEs) were observed in nearly all patients, occurring in 100% (40/40) of the triple therapy group and in 97.5% (39/40) of the dual therapy group (**Table 3**). Grade 3–4 TEAEs occurred in 15 of 40 patients (37.5%) in the triple-therapy group and 9 of 40 patients (22.5%) in the dual-therapy group. The incidence was numerically higher with triple therapy, although the difference was not statistically significant (*P* = 0.222). No treatment-related deaths or treatment discontinuations due to toxicity were recorded in either group. Overall, the safety profiles of both regimens were consistent with the established toxicity spectra of the constituent agents and no unexpected or novel adverse safety signals were identified.

**Table 3:** Treatment-emergent adverse events after matching.

Adverse events, n (%)	Any grade			Grade 1-2			Grade 3-4		
	Dual-therapy group (n = 40)	Triple-therapy group (n = 40)	*P*-value	Dual-therapy group (n = 40)	Triple-therapy group (n = 40)	*P*-value	Dual-therapy Group (n = 40)	Triple-therapy Group (n = 40)	*P*-value
Any TEAE	39 (97.5%)	40 (100.0%)	1.000	30 (75.0%)	25 (62.5%)	0.335	9 (22.5%)	15 (37.5%)	0.222
Lymphocyte count decreased	22 (55.0%)	29 (72.5%)	0.162	17 (42.5%)	29 (72.5%)	0.012	5 (12.5%)	0 (0.0%)	0.055
Platelet count decreased	17 (42.5%)	31 (77.5%)	0.003	11 (27.5%)	27 (67.5%)	<0.001	6 (15.0%)	4 (10.0%)	0.737
Anemia	15 (37.5%)	23 (57.5%)	0.117	14 (35.0%)	22 (55.0%)	0.115	1 (2.5%)	1 (2.5%)	1.000
Neutrophil count decreased	12 (30.0%)	16 (40.0%)	0.482	12 (30.0%)	16 (40.0%)	0.482	0 (0.0%)	0 (0.0%)	1.000
Abdominal pain	12 (30.0%)	14 (35.0%)	0.812	12 (30.0%)	12 (30.0%)	1.000	0 (0.0%)	2 (5.0%)	0.494
Ascites	8 (20.0%)	14 (35.0%)	0.210	6 (15.0%)	14 (35.0%)	0.069	2 (5.0%)	0 (0.0%)	0.494
GGT increased	11 (27.5%)	8 (20.0%)	0.600	9 (22.5%)	7 (17.5%)	0.781	2 (5.0%)	1 (2.5%)	1.000
ALT increased	11 (27.5%)	19 (47.5%)	0.105	10 (25.0%)	16 (40.0%)	0.232	1 (2.5%)	3 (7.5%)	0.615
Hypoalbuminemia	10 (25.0%)	16 (40.0%)	0.232	9 (22.5%)	16 (40.0%)	0.147	1 (2.5%)	0 (0.0%)	1.000
Decreased WBC count	10 (25.0%)	12 (30.0%)	0.803	10 (25.0%)	12 (30.0%)	0.803	0 (0.0%)	0 (0.0%)	1.000
AST increased	9 (22.5%)	17 (42.5%)	0.094	9 (22.5%)	14 (35.0%)	0.323	0 (0.0%)	3 (7.5%)	0.241
Fever	7 (17.5%)	12 (30.0%)	0.293	7 (17.5%)	12 (30.0%)	0.293	0 (0.0%)	0 (0.0%)	1.000
Alkaline phosphatase increased	7 (17.5%)	7 (17.5%)	1.000	6 (15.0%)	7 (17.5%)	1.000	1 (2.5%)	0 (0.0%)	1.000
Blood bilirubin increased	7 (17.5%)	7 (17.5%)	1.000	5 (12.5%)	6 (15.0%)	1.000	2 (5.0%)	1 (2.5%)	1.000
Weight loss	6 (15.0%)	10 (25.0%)	0.402	6 (15.0%)	10 (25.0%)	0.402	0 (0.0%)	0 (0.0%)	1.000
Anaphylaxis	3 (7.5%)	0 (0.0%)	0.241	3 (7.5%)	0 (0.0%)	0.241	0 (0.0%)	0 (0.0%)	1.000
Gastrointestinal hemorrhage	2 (5.0%)	5 (12.5%)	0.432	2 (5.0%)	5 (12.5%)	0.432	0 (0.0%)	0 (0.0%)	1.000
Hyperuricemia	2 (5.0%)	4 (10.0%)	0.675	2 (5.0%)	4 (10.0%)	0.675	0 (0.0%)	0 (0.0%)	1.000
Gastric ulcer	1 (2.5%)	4 (10.0%)	0.359	1 (2.5%)	4 (10.0%)	0.359	0 (0.0%)	0 (0.0%)	1.000
Proteinuria	1 (2.5%)	4 (10.0%)	0.359	1 (2.5%)	4 (10.0%)	0.359	0 (0.0%)	0 (0.0%)	1.000
Hypertension	0 (0.0%)	10 (25.0%)	0.001	0 (0.0%)	10 (25.0%)	0.001	0 (0.0%)	0 (0.0%)	1.000

TEAE, Treatment-emergent adverse event; GGT, Gamma-glutamyl transferase; ALT, Alanine aminotransferase; WBC, White blood cell; AST, Aspartate aminotransferase.

The most common any-grade TEAEs were thrombocytopenia (77.5% vs. 42.5%; *P =* 0.003), lymphopenia (72.5% vs. 55.0%; *P =* 0.162), anemia (57.5% vs. 37.5%; *P =* 0.117), and ALT elevation (47.5% vs. 27.5%; *P =* 0.105). A statistically significant between-group difference was observed in thrombocytopenia. Among the grade 1–2 events, lymphopenia (72.5% vs. 42.5%; *P =* 0.012) and thrombocytopenia (67.5% vs. 27.5%; P < 0.001) were significantly more prevalent in the triple therapy group. The overall incidence of grade 3–4 adverse events was low, with no statistically significant between-group differences for any individual grade 3–4 TEAE.

Adverse events related to TACE and anti-angiogenic therapy were more prominent in the triple therapy group. Hypertension was observed exclusively in triple therapy recipients (25.0% vs. 0%; *P =* 0.001); all cases were grade 1–2 in severity and were pharmacologically manageable. ALT elevation (47.5% vs. 27.5%; *P =* 0.105), AST elevation (42.5% vs. 22.5%; *P =* 0.094), and hypoalbuminemia (40.0% vs. 25.0%; *P =* 0.232) were more frequent in the triple therapy group; however, these differences were not statistically significant.

## Discussion

This retrospective, propensity score-matched cohort study compared the efficacy and safety of TACE combined with sintilimab and bevacizumab (triple therapy) versus sintilimab and bevacizumab alone (dual therapy) in patients with uHCC. Following PSM, the triple therapy group demonstrated a significantly prolonged median PFS (13.11 vs. 8.57 months; *P =* 0.026), a higher estimated 6-month DOR probability, and numerically higher rates of objective response and radiographic downstaging, with generally manageable toxicity but a numerically higher incidence of grade 3–4 TEAEs. Notably, the results of the exploratory prognostic scoring model demonstrated a numerically favorable PFS benefit in the intermediate-risk group under triple therapy. However, formal interaction testing did not reveal statistically significant differences in the treatment association across risk strata. To our knowledge, this is among the first studies to directly compare TACE plus sintilimab-bevacizumab with sintilimab-bevacizumab alone in patients with uHCC, providing preliminary real-world comparative evidence that warrants confirmation in prospective studies.

The combination of PD-1/PD-L1 blockade and anti-VEGF therapy has emerged as a cornerstone of first-line systemic treatment for uHCC, following the landmark IMbrave150 (16) and ORIENT-32 (17) trials that demonstrated superior OS and PFS over sorafenib with atezolizumab plus bevacizumab and sintilimab plus a bevacizumab biosimilar, respectively. However, a substantial proportion of patients do not achieve durable responses to systemic therapy alone, underscoring the rationale for incorporating locoregional modalities. TACE exerts direct cytotoxic and ischemic effects on tumor tissue while simultaneously triggering immunogenic cell death and releasing tumor-associated antigens that may potentiate systemic immune responses (18). Furthermore, TACE-induced tumor hypoxia paradoxically upregulates VEGF, creating a biological synergy with bevacizumab’s antiangiogenic mechanism and potentially amplifying the immunostimulatory milieu for PD-1 blockade (19). This mechanistic triad provides a conceptual basis for the enhanced efficacy observed in the triple therapy group in this study. Beyond clinical efficacy, the affordability of first-line immuno-combination regimens is also relevant to treatment selection. From the perspective of Chinese payers, Wen et al. (20) identified substantial differences in cost-effectiveness among available regimens, with camrelizumab plus rivoceranib representing the most cost-effective base-case strategy, followed by sintilimab plus a bevacizumab biosimilar. These findings highlight the importance of considering economic accessibility alongside efficacy when selecting systemic treatment backbones, although the analysis did not evaluate the additional costs or benefits associated with TACE.

Recent randomized trials have strengthened the evidence supporting the integration of TACE with immune-based systemic therapy. In the phase III LEAP-012 trial (18), the addition of lenvatinib and pembrolizumab to TACE significantly prolonged median PFS compared with TACE plus dual placebo (14.6 vs. 10.0 months; HR, 0.66; 95% CI, 0.51–0.84). Similarly, EMERALD-1 (15) demonstrated improved median PFS with durvalumab plus bevacizumab and TACE versus placebo plus TACE (15.0 vs. 8.2 months; HR, 0.77; 95% CI, 0.61–0.98). More recently, the randomized phase II CHANCE2005/CARES-005 trial (21) showed that TACE combined with camrelizumab and rivoceranib prolonged PFS compared with TACE alone, with consistent findings according to mRECIST. Collectively, these trials provide prospective support for combining locoregional therapy with immune checkpoint inhibition and antiangiogenic therapy. Nevertheless, these trials predominantly evaluated embolization-eligible patients without extrahepatic metastases and generally used TACE-based control regimens, whereas our study included a broader population and directly compared triple therapy with an active systemic doublet. Therefore, numerical cross-trial comparisons should be interpreted cautiously.

In the current study, the triple therapy group achieved a significantly longer median PFS of 13.11 months compared with 8.57 months in the dual therapy group (*P =* 0.026). Fixed-time analyses showed numerically higher PFS probabilities with triple therapy at 6, 9, and 12 months, with nominal P values below 0.05 at 9 and 12 months. Among responders, the estimated 6-month DOR probability was also higher with triple therapy (86.7% vs. 61.6%; *P* = 0.043), whereas the 12-month comparison did not reach statistical significance (56.1% vs. 30.8%; P = 0.066). This temporal pattern is consistent with the concept that TACE may contribute to sustained disease control through reductions in viable tumor burden and modulation of antitumor immunity (22, 23). Although the ORR (75.0% vs. 60.0%; *P =* 0.233) and DCR (95.0% vs. 82.5%; *P =* 0.154) differences did not reach statistical significance, possibly due to limited sample size following PSM, the consistent numerical advantage across all efficacy endpoints supports the biological plausibility of the triple-therapy approach. It should also be noted that patients in the triple-therapy group received more cycles of sintilimab and bevacizumab than those in the dual-therapy group. Because treatment exposure was a post-baseline, time-dependent variable, patients with longer disease control had a greater opportunity to continue systemic therapy; therefore, the greater number of treatment cycles may partly reflect, rather than solely explain, the longer PFS. Conversely, a potential contribution of prolonged systemic treatment exposure to sustained disease control cannot be excluded. These interrelated effects could not be disentangled in the present retrospective study.

The radiographic downstaging rate was numerically higher in the triple-therapy group than in the dual-therapy group (17.5% vs. 5.0%; *P* = 0.154), although the difference was not statistically significant. This observation is relevant to ongoing interest in conversion strategies for initially unresectable HCC. Zhu et al. reported an R0 resection rate of 15.9% following treatment with tyrosine kinase inhibitors and anti-PD-1 antibodies (13). Han et al. reported a higher conversion rate with bevacizumab plus sintilimab and locoregional intervention than with lenvatinib plus sintilimab and locoregional intervention (57.1% vs. 26.0%; *P* = 0.043), with conversion outcomes including surgical resection or radiofrequency ablation (10). These studies provide clinical context for investigating the potential of combination therapy to expand subsequent treatment options. However, downstaging in the present study was defined as a reduction in CNLC stage at the best radiological response assessment, rather than conversion to resectability or receipt of definitive local treatment. Therefore, these rates are not directly comparable. Because subsequent surgical resection data were not collected, whether the observed downstaging translated into an increased likelihood of resection remains unknown. Further prospective studies are warranted to evaluate radiographic downstaging and subsequent surgical outcomes separately.

An exploratory prognostic scoring model incorporating extrahepatic metastasis, tumor number >3, and Child-Pugh class B demonstrated modest discrimination (C-index = 0.639) and successfully stratified patients into low-, intermediate-, and high-risk groups with significantly divergent median PFS values of 14.46, 11.50, and 5.62 months, respectively (*P* < 0.001). The HR point estimate was numerically lowest in the intermediate-risk group, and the corresponding within-stratum comparison yielded a nominal *P* value of 0.043. However, the numerically favorable HR estimate in the intermediate-risk group should be considered hypothesis-generating, given the non-significant treatment-by-risk-group interaction (*P* for interaction = 0.879), and does not establish a subgroup-specific benefit or support treatment selection. Although the score separated PFS distributions within the derivation cohort, variable selection, coefficient estimation, and risk-group definition were all performed using the same limited dataset. Accordingly, the model and the subsequent risk-stratified analyses should be considered hypothesis-generating rather than suitable for individual treatment selection. Further evaluation in a larger independent cohort is required.

The safety profile of the triple therapy regimen was consistent with the known toxicity spectra of its constituent modalities and remained clinically manageable throughout the study. Any-grade TEAEs occurred in nearly all patients in both groups (100.0% vs. 97.5%). Grade 3–4 TEAEs were numerically more frequent with triple therapy (37.5% vs. 22.5%), although the difference was not statistically significant (*P* = 0.222). No treatment-related deaths were observed. As anticipated, TACE-associated toxicities and antiangiogenic effects were more prominent in the triple therapy group: hypertension occurred exclusively in triple-therapy recipients (25.0% vs. 0%; *P* = 0.001) and was uniformly low-grade and pharmacologically manageable. Hepatic transaminase elevations (ALT: 47.5% vs. 27.5%; AST: 42.5% vs. 22.5%) and hypoalbuminemia (40.0% vs. 25.0%) were more frequent in the triple therapy group, likely reflecting the cumulative hepatic parenchymal stress from repeated TACE procedures superimposed on systemic antiangiogenic therapy. Notably, any-grade thrombocytopenia was significantly more prevalent in the triple-therapy group, particularly at grades 1–2 (67.5% vs. 27.5%; *P* < 0.001), a finding that may reflect differential effects on bone marrow reserve or hepatic sinusoidal integrity between regimens and warrants further mechanistic investigation. Overall, the observed toxicities were generally manageable in this selected cohort. However, the numerically higher incidence of grade 3–4 TEAEs warrants caution.

This study has several limitations. First, the single-center retrospective design and individualized treatment allocation introduce selection bias and confounding by indication. BCLC-only matching left residual imbalance in other measured covariates and could not address unmeasured confounding; the findings therefore represent associations rather than causal effects. Second, although 63 PFS events were recorded, the small matched cohort (80 patients) and immature OS data limit precision and conclusions about long-term survival. Subsequent surgical data were not collected, precluding evaluation of actual resection and postoperative outcomes. Third, the heterogeneity in TACE scheduling and the number of procedures performed across patients may have introduced variability in the locoregional treatment intensity. Finally, the prognostic score was developed and evaluated in the same cohort of only 80 matched patients. Although 63 PFS events were observed, the data-driven screening process, clinically informed retention of Child–Pugh class, and use of sample-specific tertile cutoffs may have introduced optimism and overfitting. The model showed only modest discrimination, with an apparent C-index of 0.639 and an optimism-corrected C-index of 0.622. Furthermore, the risk-stratified treatment comparisons involved small and unequal subgroup sample sizes, particularly in the intermediate-risk group, and no statistically significant treatment-by-risk-group interaction was detected. Therefore, the nominally significant result within the intermediate-risk stratum may reflect sampling variability and should not be interpreted as evidence of a subgroup-specific treatment effect. This exploratory model should not be used to guide clinical treatment selection without validation in a larger independent cohort.

## Conclusions

In conclusion, TACE combined with sintilimab and bevacizumab was associated with longer PFS and greater response durability than sintilimab and bevacizumab alone. Toxicities were generally manageable, but grade 3–4 TEAEs were numerically more frequent with triple therapy, warranting cautious interpretation of its safety. An exploratory prognostic score generated hypotheses for future risk-stratified evaluation; however, the model requires independent external validation before it can be used to guide treatment selection. These findings support further prospective evaluation of adding TACE to sintilimab-bevacizumab-based treatment in appropriately selected patients with uHCC.

## Data Availability

The original contributions presented in the study are included in the article/**Supplementary Material**. Further inquiries can be directed to the corresponding author.
