# Supplementary material for: Efficacy and safety of sintilimab plus bevacizumab with or without transarterial chemoembolization for unresectable hepatocellular carcinoma: a propensity score-matched retrospective cohort study
Source: Front Immunol. 2026 Oct 26;17:1928849. doi: 10.3389/fimmu.2026.1928849 (PMC13745478; doi:10.3389/fimmu.2026.1928849)
Supplement: Supplementary file 1 [file Table1.docx]

**Title:** Efficacy and Safety of Sintilimab Plus Bevacizumab With or Without Transarterial Chemoembolization for Unresectable Hepatocellular Carcinoma: A Propensity Score-Matched Retrospective Cohort Study

**Authors:**

Zizhuo Wang^1,2,3†^, MD, Bailu Wu^1,2,3†^, PhD, Chunshu Wu^1,2,3†^, MD, Hongquan Wu^1,2,3^, MD, Shihao Zhao^1,2,3^, MD, Shuwen Ye^1,2,3^, MD, Xiaodong Li^1,2,3^, MD, Pengchao Zhan^4^, MD, PhD, Xin Li^1,2,3^, MD, Zhen Li^1,2,3*^, MD, PhD

**Affiliations:**

^1^ Department of Interventional Radiology, the First Affiliated Hospital of Zhengzhou University, No.1, East Jian She Road, Zhengzhou 450052, Henan Province, China

^2^ Henan Provincial Minimally Invasive Interventional Tumor Engineering and Technology Research Center

^3^ Key Laboratory of Innovative and Translational Interventional Oncology Technology in Zhengzhou

^4^ Department of Radiology, the Third People's Hospital of Henan Province, No. 198, Funiu Road, Zhengzhou 450000, Henan Province, China

^†^ These authors contributed equally

^*^ The corresponding author

**Corresponding author:**

Zhen Li, MD, PhD,

Department of Interventional Radiology, the First Affiliated Hospital of Zhengzhou University, No.1, East Jian She Road, Zhengzhou 450052, Henan Province, China

Telephone: +86 15837192255

Fax number: +86 0371 6686 2162

E-mail: [lzlyct620@163.com](mailto:lzlyct620@163.com)

**Contents:**

Supplementary Method 1. Detailed statistical procedures and prognostic score development

Supplementary Table 1. Patient Baseline Characteristics Before Matching

Supplementary Table 2. Covariate balance before and after propensity score matching

Supplementary Table 3. Treatment Cycles After Matching

Supplementary Table 4. Predictors of Objective Response Rate After Matching (Logistic Regression)

Supplementary Table 5. Predictors of Progression-Free Survival After Matching (Cox Regression)

Supplementary Figure 1. Love plot of covariate balance before and after propensity score matching.

**Supplementary Method 1. Detailed statistical procedures and prognostic score development**

Categorical variables are presented as frequencies with percentages. Continuous data were expressed as medians with ranges. The Student’s t-test, Wilcoxon rank-sum test, Pearson’s Chi-squared test, and Fisher’s exact test were used to compare variables, depending on the nature of the data. Propensity score matching (PSM) was performed using the MatchIt package (version 4.5.2) in R. A 1:1 nearest-neighbor matching algorithm was applied without replacement, with a caliper of 0.02 standard deviations of the propensity score. Propensity scores were estimated using baseline BCLC stage as the sole covariate. Given the limited sample size, this parsimonious matching strategy was chosen because BCLC stage is clinically relevant to both prognosis and treatment selection in HCC. CNLC stage was assessed as part of the covariate balance diagnostics before and after matching. Covariate balance before and after matching was assessed using absolute standardized mean differences. An absolute SMD of 0.10 was used as a commonly adopted reference value for identifying potential residual imbalance and was interpreted as a descriptive rather than an absolute pass-or-fail criterion. PFS, OS, TTR, and DOR with associated 95% confidence intervals (CIs) were estimated using the Kaplan-Meier method and compared using log-rank tests. Patients who maintained no events at the time of analysis were censored at the last imaging evaluation. Fixed-time PFS (6, 9, and 12 months) and DOR (6 and 12 months) probabilities were estimated using the Kaplan-Meier method and compared using two-sided Wald tests on the log[−log(S(t))] scale with Greenwood variances, with no matched-pair covariance or multiplicity adjustment.

A multivariable Cox proportional hazards model was used to identify independent prognostic factors by entering the associated variables (*P* ≤ 0.10) in the univariate analysis. Hazard ratios (HRs) and 95% CI for the PFS subgroup analyses were estimated using Cox proportional hazards models, with the corresponding P values calculated using Wald tests. A Cox model incorporating the selected variables was subsequently refitted, and its regression coefficients were used to calculate the linear predictor. Patients were divided into low-, intermediate-, and high-risk groups using sample-specific tertiles of the linear predictor. Model discrimination was quantified using Harrell’s C-index. Internal validation of the fixed three-variable model was performed using 1,000 bootstrap resamples to estimate optimism, from which an optimism-corrected C-index was calculated. PFS distributions across the prognostic risk groups were estimated using the Kaplan-Meier method and compared using the log-rank test. Within each risk stratum, the effect of triple therapy versus dual therapy was estimated using univariable Cox proportional hazards models, with results reported as HR and 95% CI. The corresponding P values were calculated using Wald tests. The model and the subsequent risk-stratified analyses were considered exploratory.

All statistical tests were two-tailed, and statistical significance was set at *P* < 0.05. Analyses were performed using the R software (version 4.5.2) and GraphPad Prism 10.0 (GraphPad Software, La Jolla, CA, USA).

**Supplementary Table 1. Patient Baseline Characteristics Before Matching**

| Characteristics, n (%) | Dual-therapy group  (n = 84) | Triple-therapy group  (n = 40) | *P*-value |
| --- | --- | --- | --- |
| Gender |  |  | 0.060 |
| Female | 13 (15.5%) | 12 (30.0%) |  |
| Male | 71 (84.5%) | 28 (70.0%) |  |
| Age, years |  |  | 0.581 |
| <50 | 16 (19.0%) | 6 (15.0%) |  |
| ≥50 | 68 (81.0%) | 34 (85.0%) |  |
| PVTT |  |  | <0.001 |
| Vp 0 | 59 (70.2%) | 9 (22.5%) |  |
| Vp 1 | 0 (0.0%) | 3 (7.5%) |  |
| Vp 2 | 10 (11.9%) | 10 (25.0%) |  |
| Vp 3 | 8 (9.5%) | 8 (20.0%) |  |
| Vp 4 | 7 (8.3%) | 10 (25.0%) |  |
| HVTT |  |  | 0.005 |
| absent | 77 (91.7%) | 29 (72.5%) |  |
| present | 7 (8.3%) | 11 (27.5%) |  |
| Extrahepatic spread |  |  | 0.011 |
| absent | 73 (86.9%) | 27 (67.5%) |  |
| present | 11 (13.1%) | 13 (32.5%) |  |
| CNLC stage |  |  | <0.001 |
| 1a | 7 (8.3%) | 0 (0.0%) |  |
| 1b | 15 (17.9%) | 0 (0.0%) |  |
| 2a | 6 (7.1%) | 1 (2.5%) |  |
| 2b | 20 (23.8%) | 4 (10.0%) |  |
| 3a | 25 (29.8%) | 22 (55.0%) |  |
| 3b | 11 (13.1%) | 13 (32.5%) |  |
| BCLC |  |  | <0.001 |
| A | 23 (27.4%) | 0 (0.0%) |  |
| B | 25 (29.8%) | 5 (12.5%) |  |
| C | 36 (42.9%) | 35 (87.5%) |  |
| Tumor size^*^, cm | 7.60 (1.20–18.60) | 7.55 (2.40–14.70) | 0.685 |
| Tumor number |  |  | 0.633 |
| ≤3 | 50 (59.5%) | 22 (55.0%) |  |
| >3 | 34 (40.5%) | 18 (45.0%) |  |
| Up-to-7 |  |  | 0.407 |
| ≤7 | 13 (15.5%) | 4 (10.0%) |  |
| >7 | 71 (84.5%) | 36 (90.0%) |  |
| AFP, ng/mL |  |  | 0.321 |
| <400 | 56 (66.7%) | 23 (57.5%) |  |
| ≥400 | 28 (33.3%) | 17 (42.5%) |  |
| Etiology |  |  | 0.709 |
| HCV | 11 (13.1%) | 3 (7.5%) |  |
| HBV | 69 (82.1%) | 35 (87.5%) |  |
| None | 4 (4.8%) | 2 (5.0%) |  |
| ALBI grade |  |  | 0.082 |
| 1 | 38 (45.2%) | 10 (25.0%) |  |
| 2 | 44 (52.4%) | 29 (72.5%) |  |
| 3 | 2 (2.4%) | 1 (2.5%) |  |
| Child-Pugh |  |  | 0.375 |
| A | 61 (72.6%) | 32 (80.0%) |  |
| B | 23 (27.4%) | 8 (20.0%) |  |
| ECOG PS |  |  | 0.871 |
| 0 | 37 (44.0%) | 17 (42.5%) |  |
| 1 | 47 (56.0%) | 23 (57.5%) |  |
| NLR^*^ | 2.46 (0.66–35.53) | 2.70 (0.76–9.06) | 0.667 |

^*^Median (range). PVTT, Portal vein tumor thrombus; HVTT, Hepatic vein tumor thrombus; CNLC, China Liver Cancer Staging; BCLC, Barcelona Clinic Liver Cancer; AFP, Alpha-fetoprotein; ALBI, Albumin-Bilirubin grade; ECOG PS, Eastern Cooperative Oncology Group Performance Status; NLR, Neutrophil-to-Lymphocyte Ratio

**Supplementary Table 2. Covariate balance before and after propensity score matching**

| Variable | SMD before | SMD after |
| --- | --- | --- |
| Gender (Male) | 0.317 | 0.382 |
| Age (≥50 years) | 0.113 | 0.350 |
| PVTT |  |  |
| Vp0 | 1.143 | 0.419 |
| Vp1 | 0.285 | 0.285 |
| Vp2 | 0.302 | 0.058 |
| Vp3 | 0.262 | 0.000 |
| Vp4 | 0.385 | 0.173 |
| HVTT (Present) | 0.429 | 0.224 |
| Extrahepatic spread (Present) | 0.414 | 0.107 |
| CNLC stage |  |  |
| 1a | 0.366 | 0.000 |
| 1b | 0.566 | 0.000 |
| 2a | 0.297 | 0.000 |
| 2b | 0.46 | 0.000 |
| 3a | 0.507 | 0.101 |
| 3b | 0.414 | 0.107 |
| BCLC stage |  |  |
| A | 0.746 | 0.000 |
| B | 0.522 | 0.000 |
| C | 1.35 | 0.000 |
| Tumor size | 0.099 | 0.325 |
| Tumor number (>3) | 0.091 | 0.201 |
| Up-to-7 (>7) | 0.183 | 0.167 |
| AFP (≥400 ng/mL) | 0.185 | 0.101 |
| Etiology |  |  |
| HCV | 0.212 | 0.095 |
| HBV | 0.162 | 0.000 |
| None | 0.011 | 0.115 |
| ALBI grade |  |  |
| 1 | 0.467 | 0.346 |
| 2 | 0.451 | 0.336 |
| 3 | 0.008 | 0.000 |
| Child-Pugh class (B) | 0.185 | 0.375 |
| ECOG PS (1) | 0.031 | 0.152 |
| NLR | 0.249 | 0.002 |

AFP, Alpha-fetoprotein; ALBI, Albumin–bilirubin; BCLC, Barcelona Clinic Liver Cancer; CNLC, China Liver Cancer; ECOG PS, Eastern Cooperative Oncology Group performance status; HBV, Hepatitis B virus; HCV, Hepatitis C virus; HVTT, Hepatic vein tumor thrombus; NLR, Neutrophil-to-lymphocyte ratio; PVTT, Portal vein tumor thrombus; SMD, Standardized mean difference

**Supplementary Table 3. Treatment Cycles After Matching**

| Treatment | Dual-therapy group (n = 40) | Triple-therapy group (n = 40) | *P*-value |
| --- | --- | --- | --- |
| TACE | NA | 2.0 (1.0–3.0) | - |
| Sintilimab | 8.0 (2.0–33.0) | 13.0 (2.0–33.0) | 0.011 |
| Bevacizumab | 6.5 (2.0–22.0) | 13.0 (2.0–33.0) | <0.001 |

TACE, Transarterial chemoembolization. Data are presented as median (range)

**Supplementary Table 4. Predictors of Objective Response Rate After Matching (Logistic Regression)**

| Variable | Univariable analysis | | |  | Multivariable analysis | | |
| --- | --- | --- | --- | --- | --- | --- | --- |
|  | OR | 95% CI | *P*-value |  | OR | 95% CI | *P*-value |
| Gender (male vs female) | 0.83 | 0.24–2.58 | 0.760 |  |  |  |  |
| Age (≥50 vs <50) | 0.83 | 0.24–2.58 | 0.760 |  |  |  |  |
| Vascular invasion  (present vs absent) | 1.16 | 0.38–3.33 | 0.783 |  |  |  |  |
| Extrahepatic spread  (present vs absent) | 1.25 | 0.45–3.70 | 0.677 |  |  |  |  |
| BCLC (C vs B) | 1.45 | 0.34–5.62 | 0.590 |  |  |  |  |
| Tumor number (>3 vs ≤3) | 0.23 | 0.08–0.63 | 0.006 |  | 0.25 | 0.08–0.71 | 0.011 |
| Up-to-seven (>7 vs ≤7) | 1.04 | 0.14–5.73 | 0.964 |  |  |  |  |
| AFP (≥400 vs <400) | 0.29 | 0.10–0.75 | 0.013 |  | 0.32 | 0.11–0.87 | 0.028 |
| Etiology (HBV vs others) | 2.33 | 0.59–9.23 | 0.216 |  |  |  |  |
| Child-Pugh (B vs A) | 1.04 | 0.37–3.12 | 0.9361 |  |  |  |  |
| ECOG PS (1 vs 0) | 0.60 | 0.21–1.58 | 0.311 |  |  |  |  |
| ALBI grade (2-3 vs 1) | 1.62 | 0.65–4.12 | 0.305 |  |  |  |  |
| NLR | 1.15 | 0.93–1.47 | 0.223 |  |  |  |  |

BCLC, Barcelona Clinic Liver Cancer; AFP, Alpha-fetoprotein; HBV, Hepatitis B virus; ECOG PS, Eastern Cooperative Oncology Group Performance Status; ALBI, Albumin-Bilirubin grade; NLR, Neutrophil-to-Lymphocyte Ratio; OR, Odds Ratio; CI, Confidence Interval

**Supplementary Table 5. Predictors of Progression-Free Survival After Matching (Cox Regression)**

| Variable | Univariable analysis | | |  | Multivariable analysis | | |
| --- | --- | --- | --- | --- | --- | --- | --- |
|  | HR | 95% CI | *P*-value |  | HR | 95% CI | *P*-value |
| Gender (male vs female) | 2.01 | 0.99–4.09 | 0.055 |  | 0.35 | 0.06–2.16 | 0.259 |
| Age (≥50 vs <50) | 0.59 | 0.33–1.05 | 0.073 |  | 1.00 | 0.52–1.89 | 0.991 |
| Vascular invasion  (present vs absent) | 0.67 | 0.38–1.16 | 0.152 |  |  |  |  |
| Extrahepatic spread  (present vs absent) | 2.06 | 1.21–3.51 | 0.008 |  | 1.85 | 1.04–3.28 | 0.035 |
| BCLC (C vs B) | 0.79 | 0.39–1.61 | 0.520 |  |  |  |  |
| Tumor number (>3 vs ≤3) | 1.63 | 0.99–2.70 | 0.056 |  | 1.95 | 1.12–3.4 | 0.019 |
| Up-to-seven (>7 vs ≤7) | 1.23 | 0.44–3.38 | 0.6955 |  |  |  |  |
| AFP (≥400 vs <400) | 1.06 | 0.64–1.76 | 0.821 |  |  |  |  |
| Etiology (HBV vs others) | 1.37 | 0.59–3.20 | 0.462 |  |  |  |  |
| Child-Pugh (B vs A) | 1.93 | 1.14–3.28 | 0.015 |  | 1.71 | 0.98–2.99 | 0.060 |
| ECOG PS (1 vs 0) | 1.69 | 1.00–2.86 | 0.051 |  | 1.62 | 0.91–2.88 | 0.102 |
| ALBI grade (2-3 vs 1) | 0.96 | 0.57–1.61 | 0.876 |  |  |  |  |
| NLR | 0.96 | 0.86–1.08 | 0.509 |  |  |  |  |

Variables with a univariable *P* value ≤0.10 were entered into the multivariable model. Because sex violated the proportional hazards assumption, it was modeled using a time-dependent coefficient. BCLC, Barcelona Clinic Liver Cancer; AFP, Alpha-fetoprotein; HBV, Hepatitis B virus; ECOG PS, Eastern Cooperative Oncology Group Performance Status; ALBI, Albumin-Bilirubin grade; NLR, Neutrophil-to-Lymphocyte Ratio; HR, Hazard Ratios; CI, Confidence Interval

**Supplementary Figure 1.** Love plot of covariate balance before and after propensity score matching. The vertical dashed line at an absolute SMD of 0.10 represents a commonly used reference value for identifying potential residual imbalance. SMDs were interpreted continuously rather than as an absolute criterion for successful matching.
